# Evaluation of a Follow-Up Health Consultation Program for Patients with Coronavirus Disease 2019 in Korea: Using the Context–Input–Process–Product Model

**DOI:** 10.3390/ijerph19137996

**Published:** 2022-06-29

**Authors:** Keun-Mi Lee, Hae-Jin Ko, Geon Ho Lee, Yun-A Kim, Seung-Pil Jung, A-Sol Kim

**Affiliations:** 1 Department of Family Medicine, Yeungnam University Medical Center, College of Medicine, Yeungnam University, Daegu 42415, Korea; kmlee@yu.ac.kr (K.-M.L.); spjung@ynu.ac.kr (S.-P.J.); 2 Department of Family Medicine, School of Medicine, Kyungpook National University, Kyungpook National University Hospital, Daegu 41944, Korea; liveforme@knu.ac.kr; 3 Department of Family Medicine, Daegu Catholic University School of Medicine, Daegu 42472, Korea; totoslee@cu.ac.kr (G.H.L.); yakim@cu.ac.kr (Y.-A.K.); 4 Department of Family Medicine, School of Medicine, Kyungpook National University, Kyungpook National University Chilgok Hospital, Daegu 41404, Korea

**Keywords:** COVID-19, pandemic, consultation, context–input–process–product, evaluation

## Abstract

Beyond physical pain, patients with coronavirus disease 2019 (COVID-19) experience psychological anxiety during and after quarantine, often facing negative perceptions when returning to their communities. This study evaluated a health consultation program in Korea for post-quarantine patients with COVID-19, designed to help them return to their communities. The program was conducted from 9 March to 5 June 2020, in Daegu, Korea. In total, 20 doctors and 504 recovered patients were surveyed via questionnaire. The survey, comprising open-ended questions rated on a five-point Likert scale, was based on the Context–Input–Process–Product program evaluation model. Reliability was assessed, and descriptive statistics were obtained. A regression analysis was performed on factors affecting product (output) areas. As a main result, both doctors and recovered patients evaluated the program positively. The mean program effectiveness score was 4.00 in the doctors’ evaluations and 3.95 in the patients’ evaluations. Moreover, the input and process variables affected the product. This first-of-its-kind health consultation program proved to be an effective practical intervention for patients returning to the community after an infectious disease; it also highlights aspects that could increase satisfaction in systemized subsequent programs, with input and process areas for patients and doctors.

## 1. Introduction

In December 2019, the first case of coronavirus disease 2019 (COVID-19) was reported in Wuhan, China, as pneumonia with an unknown cause. Subsequently, severe acute respiratory syndrome coronavirus 2 (SARS-CoV-2) spread rapidly, with the World Health Organization declaring it a pandemic in March 2020. Social distancing measures were enacted worldwide [1], aimed at limiting contact among citizens to reduce disease propagation [2,3].

Korea has maintained a social distancing policy since the onset of the pandemic. After November 2021, the Korean government sought approaches to slowly phase out social distancing. However, due to continued serious disease transmission, the government had to maintain its social distancing policy. Korea’s policy requires that those infected self-isolate for at least 10 days, even if asymptomatic. The isolation period is longer for those with symptoms, especially if the symptoms are persistent. Studies show that, in addition to the physical symptoms of COVID-19, patients also experience psychological symptoms, such as anxiety, due to the isolation [4]. Some also fear social stigma [5]. Thus, when patients return to their normal lives, many face additional difficulties.

In Daegu, Korea, the spread of SARS-CoV-2 was rampant after March 2020, and certain religious groups were particularly affected [6]. The rapid spread of COVID-19 was followed by widespread anxiety in the community [7]. Patients who re-entered society after isolation faced ongoing physical and psychological issues, such as negative perceptions or stigma—especially those belonging to the religious groups—and some even felt condemned by society [8,9]. This created a critical need for healthcare programs that facilitate the reintegration of these patients into society.

To address this issue, the Daegu-Gyeongbuk branch of the Korean Academy of Family Medicine developed a Follow-up Health Consultation Program, operated by Daegu City and the Daegu Medical Association, for patients who had recovered from COVID-19 [10]. Twenty family doctors were recruited as volunteer consultants to assist patients in the community. The program was the first of its kind worldwide, and its usefulness is yet to be evaluated. Therefore, this study aims to understand the program’s process and determine its value and ease of use. This evaluation will enable us to identify areas of improvement for subsequent programs and gain feedback regarding any necessary adjustments to the program’s priorities [11].

Several models for program evaluation exist; these include the Context–Input–Reaction–Output model, the Kirkpatrick model, the Logic model, and the Context–Input–Process–Product (CIPP) model. Of these, the CIPP model is frequently recommended for evaluating educational programs. Stufflebeam discussed the potential of the CIPP evaluation method in 1971 [12]. The CIPP classifies a program according to four areas: context, inputs, processes, and products [13,14]. The usefulness of this model lies in its ability to not only assess the value of a program but to also provide concrete information about its purpose, resources, and procedures. Thus, it has the advantage of providing a systematic analysis of program performance [15]. Therefore, we chose the CIPP model to evaluate the health consultation program for patients who have recovered from COVID-19 in Korea.

## 2. Methods and Materials

### 2.1. The Follow-up Health Consultation Program

The Follow-up Health Consultation Program goals included: identifying the physical symptoms that remained after the patients emerged from quarantine, arrangements for the treatment of these symptoms in clinics or hospitals, and addressing the psychological problems associated with the patients’ return to their communities. The program and its protocol are described in detail in Kim et al.’s study [10].

As a part of the program, doctors call patients to check on their physical health and provide them with health education and psychological counseling. They also advise patients if additional treatment is needed and coordinate hospitals visits. The patients in the program receive educational brochures with guidelines related to their recovery, face masks, and hand sanitizers.

The program involved 20 consulting doctors and 1679 patients and was conducted from 9 March to 5 June 2020. A total of 75 patients—who submitted incorrect mobile numbers, did not respond to doctors’ calls, or had been admitted to hospitals two or more times—were excluded.

### 2.2. Program Evaluation Questionnaire

**Figure 1:**
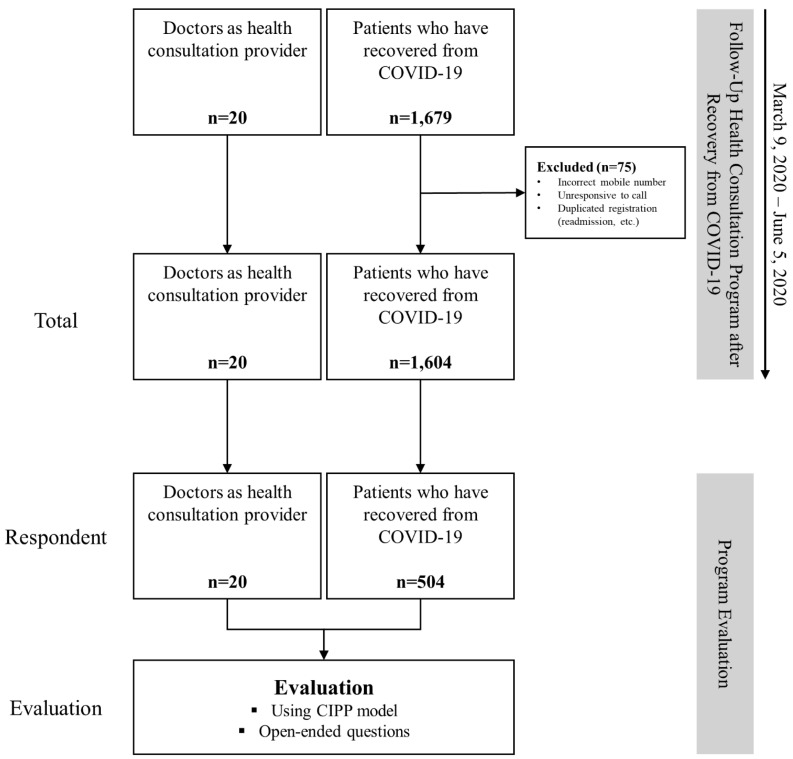
Flowchart of the study. A survey was conducted for program evaluation of 20 doctors and 1604 patients who participated in the Follow-Up Health Consultation Program. All 20 doctors (100%) and 504 of the 1604 patients (31.4%) responded to the questionnaire. COVID-19: coronavirus disease 2019; CIPP: Context–Input–Process–Product.

After the program ended, evaluation questionnaire surveys were sent to all participating patients and doctors. The schematic flow of the study is presented in Figure 1. We were able to gather complete data from all 20 doctors (100%) and 504 patients (31.4%). The study was reviewed and approved by the Institutional Review Board of Yeungnam University Medical Center (No. 2021-08-010). The need for informed consent was waived, as this is a retrospective analysis.

The doctors’ survey comprised 18 items—of which six were open-ended questions—assessed using a five-point Likert scale from “1 = strongly disagree” to “5 = strongly agree”; a higher score reflects greater satisfaction with the program. The structured questionnaires were sent to and returned by the doctors via e-mail (Supplementary Materials). Table 1 shows the content areas in the doctors’ CIPP evaluation survey. Six researchers independently extracted content from the answers to the open-ended questions, and the content was subsequently integrated. Overlapping content was identified and defined once all six authors agreed.

The patients’ survey included 16 items—of which three were open-ended questions—assessed using a five-point Likert scale. As with the doctors’ scores, a higher score denoted greater satisfaction with the program. The patients received a link to the online survey (Google Survey) through text messages sent after the consultation period ended (Supplementary Materials). Table 2 shows the content areas in the patients’ CIPP evaluation survey. The analysis of the open-ended responses was addressed in the same way as that of the doctors.

### 2.3. Statistical Analysis

We assessed reliability using Cronbach’s alphas for each area in both questionnaires. Descriptive statistics (mean and standard deviation [SD]) were used to summarize the data pertaining to each item. A multiple regression analysis was performed using the context, input, and process evaluation results of patient data as independent variables to determine the factors affecting the evaluation of the product (output) area. Open-ended questions were assessed using qualitative analysis. All statistical analyses were performed using IBM SPSS Statistics version 26.0 software (IBM Corp., Armonk, NY, USA). A *p*-value below 0.05 was considered statistically significant.

## 3. Results

### 3.1. Demographics

The mean age, sex, and mean work experience of the doctors are presented in Table 3 below. Six doctors worked in teaching hospitals, three in local hospitals, two in group practices, and nine in independent practices.

The mean age of the patients was 40.79 years. Of them, 77.2% had no comorbid diseases, while 7.8% had been quarantined for less than one week, and 16.2% for more than one month (Table 3).

### 3.2. Doctors’ Evaluations

In the doctors’ evaluations, all areas received a mean score of three or more out of five. Each evaluation area received a high average score: 4.30 for the validity of the goal in the context area, 4.20 for the usefulness of support items in the input area, 4.00 for the responsiveness to calls in the process area, and 4.00 for satisfaction with the program in the product (output) area. In the process sub-areas, the degree of interference with work and personal time scored higher than three, indicating that the doctors felt somewhat inconvenienced by their participation in the program (Table 4).

### 3.3. Patients’ Evaluations

All areas received an average score of three or higher out of five in the patients’ evaluations. The mean score for being informed of the program’s purpose in the context area was 4.22, for the expertise of doctors in the input area was 4.10, for easy-to-understand explanations from doctors in the process area was 4.24, and for satisfaction with phone consultations in the product (output) area was 4.10. The lowest mean score (3.64) was in the process area, which measured whether the support provided was timely (Table 5).

### 3.4. Correlation of Evaluation Areas

A multiple regression analysis was performed to identify the CIPP areas associated with the patients’ satisfaction with the program. The model was statistically significant (R^2^ = 0.71, *p* < 0.001). The scores of the input and process variables were significantly associated with program satisfaction, while context area scores were not (Table 6).

### 3.5. Open-Ended Responses

When asked about their opinions of the program, the doctors’ responses included: “I was able to understand the physical and mental pain of the patients who suffered in this pandemic”, and “I was able to increase my understanding of COVID-19 while conducting the consultation”. Positive statements were: “It helped a lot as a doctor”, and “Patients showed better-than-expected responses”. Negative opinions included: “There was a limit to solving the patient’s problem with only phone consultations and no face-to-face treatment”, and “There was limited understanding of new infectious diseases”. In addition, one doctor stated: “Some patients called me late at night or wanted too many consultations”, and “It was difficult to consult with not only the patient but also their family members”.

When patients were asked about their opinions of the program, examples of positive responses were: “My anxiety was resolved through consultations with doctors”, and “The masks and educational brochures were useful”. Negative opinions included: “It took a long time for the support items to be delivered”, and “The number of consultations seemed insufficient”.

## 4. Discussion

This study applied the CIPP model to evaluate the Follow-up Health Consultation Program for COVID-19 patients administered post quarantine—the first of its kind in Korea—via doctor and patient questionnaires designed to evaluate the program. The results show a mean score of three or higher for all CIPP areas.

The doctors positively evaluated the program in all four CIPP areas. The validity of the goal in the context area and the usefulness of patient items in the input area were evaluated favorably. Doctors clearly understood the goals of the program, believed that the program progressed appropriately, and judged the input resources to be useful. Regarding the product (output), program usefulness and satisfaction were also rated favorably. However, the mean score for the degree to which participation in the program interfered with their work or personal time was also high. This implies that doctors perceived some disruption in their daily lives because of their participation in the program, primarily because they had to make time for phone consultations during or after work, while also completing their usual job tasks. Regarding phone consultations, 60% of the calls took between 11 to 20 min, exceeding the usual time allocated for doctors’ outpatient clinic consultations [16]. Additionally, as the doctors participated in the program voluntarily, the lack of any compensation may have influenced this aspect of their evaluation. In their open-ended responses, they also mentioned dissatisfaction with late-night or frequent consultation requests. To address this in the future, specific consultation times should be predefined and enforced, and the number of consultations per day limited.

In the context area, doctors evaluated the possibility of achieving the program’s goals in a very positive light. However, in the product (output) area, goal achievement scored lower than in other areas, such as program satisfaction. This may be due to the lack of face-to-face consultations to identify patients’ physical problems, which was a goal of the program. Doctors expressed dissatisfaction with the limitations of distance consultations and their own limited understanding of the new infectious disease. According to a United States study on COVID-19, telemedicine has been shown to be useful in infectious disease pandemic situations; however, it has limited effectiveness when there is a need to address physical symptoms [17]. In future programs, patients should fully understand that phone consultations cannot solve all their medical problems, and goals should be set reflecting this limitation.

The patients positively evaluated all program areas. Specifically, high scores were assigned to the expertise of doctors, easy-to-understand explanations, and patients’ satisfaction with phone consultations. Furthermore, the mean scores for program effectiveness and goal achievement were 3.95 and 3.91, respectively, indicating a high overall evaluation of the program. However, the mean score for whether support items were provided in a timely manner was low, at 3.64. As support items were delivered through the postal system, there was a two- to three-day delay due to insufficient administrative support, especially as the number of patients increased. In future programs, private or alternative systems should be considered for delivering support items in a timely manner. The patients’ open-ended responses indicated that, overall, their anxiety was resolved, and their concerns addressed, confirming the program’s usefulness.

The psychological difficulties of those infected with COVID-19 have been recognized as an important issue globally [18,19,20,21]. Moreover, health practitioners and researchers agree on the need for systematic psychological interventions for infectious diseases in general [22]. In this context, our finding that the survey evaluations confirmed that patients’ anxiety had been relieved and stabilized during this health consultation program is significant. Nevertheless, patients felt that the number of consultations was insufficient. This conflicted with the doctors’ opinions, thereby indicating a need for more specific and reasonable standards for the number of consultations in future programs.

In terms of reducing post-infection social stigma or negative perceptions, no significant effects were observed. One possible reason may be the lack of face-to-face consultations. Moreover, the evaluation did not allow us to assess individuals’ specific social situation, such as psychiatric medical history and economic condition. Additionally, many of the patients surveyed belonged to specific religious groups. This factor should be considered in future programs when setting goals and establishing processes.

The regression analysis determined which CIPP area variables affected a patient’s satisfaction with the program. Program satisfaction was high when the input and process variables were evaluated positively. Generally, in the case of education or improvement programs, if satisfaction with the resources and operational process is low, the satisfaction with the program will also be low [23,24]. Therefore, to increase program satisfaction, the input and process variables must be well configured. In this program, patient’s satisfaction with the timing of the support items was somewhat low; therefore, intervention timing should be prioritized in future programs.

In this study, the context variables did not affect program satisfaction. In contrast to the doctors’ surveys, patients evaluated only “being well-informed of the program’s purpose” as a context variable—and very positively so—such that there was no statistical significance. Nonetheless, if the context is not clearly presented in the program, it can significantly reduce the likelihood of achieving its goals [25]. Thus, the focus should be on the evaluation of the context variables by the participants, in line with the input and process variables.

Despite its clear contributions, several limitations of the study warrant further comment. First, as the study considered a single city dominated by a specific religious group, it is difficult to generalize the results. However, we believe that our study’s findings are valuable, as the operation of such a program and its evaluation are necessary to address the emotional distress caused by an infectious disease such as COVID-19. Second, not all questionnaire items in the study were part of the CIPP evaluation. However, if a questionnaire is too closely based on an evaluation method, or if several different surveys are conducted, the response rate may decrease; this is especially true for patients with COVID-19 who may feel overburdened, given that they are experiencing the physical effects of the disease as well as the psychological effects of social issues. Thus, questions outside the CIPP evaluation areas were added to achieve our objectives. In the future, the questionnaire design could be improved by including only a manageable number of questions based solely on the evaluation model. Third, when researchers interpret the opinions expressed in open-ended responses, there is a risk of subjectivity. However, each of the six authors independently extracted the opinions and reached an agreement, after which the results were integrated; thus, we attempted to minimize the influence of each researcher’s subjective judgment.

Despite these limitations, this novel study contributes to the literature by systematically evaluating the first post-quarantine health consultation program for patients with COVID-19. Furthermore, as the program was conducted in Daegu, where the first serious outbreak occurred in Korea in 2020, the program represents a positive and proactive move by the government.

## 5. Conclusions

Ultimately, the health consultation program in Korea for recovered patients with COVID-19—who continue to have physical, psychological, and social problems after isolation—was deemed effective, useful, and highly satisfactory. Accordingly, developing and operating a well-structured systematic follow-up health consultation program should be considered an effective intervention for patients recovering from infectious diseases, especially those who are likely to suffer psychological problems due to social isolation.

## Figures and Tables

**Table 1:** Doctors’ CIPP questionnaire content areas in the Follow-up Health Consultation Program evaluation.

CIPP Area	Content	Number of Questions	Reliability *
Context	Goal	3	0.859
Input	Resources (e.g., medical resources)	2	0.813
Process	Operation of program	4	0.811
	Burden of work		
Product (Output)	Effectiveness	3	0.893
	Satisfaction		
	Impact of the program		
	Positive and negative outcomes	Open-ended questions	

* Cronbach’s α. CIPP: Context–Input–Process–Product.

**Table 2:** Patients’ CIPP questionnaire content areas in the Follow-up Health Consultation Program evaluation.

CIPP Area	Content	Number of Questions	Reliability *
Context	Goal	1	N/A
Input	Resources (e.g., medical resources)	3	0.716
Process	Operation of program	4	0.859
Product (Output)	Satisfaction	5	0.807
	Effectiveness		
	Impact of program		
	Positive and negative outcomes	Open-ended questions	

* Cronbach’s α. CIPP: Context–Input–Process–Product.

**Table 3:** Descriptive statistics of participating doctors and patients.

Doctors (n = 20)	Mean ± SD/n (%)
Age	45.10 ± 5.73
Work experience, years	14.15 ± 6.58
Sex	
Male	14 (70)
Female	6 (30)
Workplace/type	
Teaching hospital	6 (30.0)
Local hospital	3 (15.0)
Group practice	2 (10.0)
Individual practice	9 (45.0)
Phone consultation time, min	
<10	8 (40.0)
11–20	12 (60.0)
21–30	0 (0.0)
>31	0 (0.0)
**Patients (n = 504)**	
Age	40.79 ±14.95
Sex	
Male	121 (29.7)
Female	287 (70.3)
Family composition	
Single	83 (20.3)
Married	70 (17.2)
≥2 generations together	190 (46.6)
Other	65 (15.9)
Comorbidities	
None	315 (77.2)
1	70 (17.2)
>2	23 (5.6)
Hospitalization	
Inpatient facility	207 (50.7)
Medical institution	212 (52.0)
Intensive care unit	12 (2.9)
Other	22 (16.2)
Quarantine period, days	
1–7	32 (7.8)
8–14	109 (26.7)
15–21	125 (30.6)
22–28	76 (18.6)
>29	66 (16.2)

SD: standard deviation.

**Table 4:** Sub-area evaluation of the Follow-up Health Consultation Program by doctors.

Area	Content	Evaluation items	Score *
Context	Goal	Clarity of goal	4.00 ± 0.89
		Validity of goal	4.30 ± 0.56
		Possibility of achieving goals	3.75 ± 0.43
Input	Medical resources	Adequacy of allotment	3.65 ± 0.91
	Supplies	Usefulness of support items (educational brochure, face mask)	4.20 ± 0.75
Process	Operation of program	Responsiveness to calls	4.00 ± 0.71
		Responsiveness to consultations	3.75 ± 0.62
	Impediments	Degree of disruption to main work	3.75 ± 0.62
		Degree of disturbance outside of work	3.35 ± 0.96
Product (output)	Effectiveness	Program effectiveness	4.00 ± 0.45
	Satisfaction	Satisfaction with the program as a doctor	4.00 ± 0.55
	Impact of program	Achievement of program goals	3.80 ± 0.60

* Mean ± standard deviation.

**Table 5:** Sub-area evaluation of the Follow-up Health Consultation Program by recovered patients.

Area	Content	Evaluation Items	Score *
Context	Goal	Informed of the program’s purpose	4.22 ± 0.77
Input	Medical resources	Expertise of the doctors	4.10 ± 0.83
	Supplies	Usefulness of support items (educational brochure)	3.74 ± 0.95
		Usefulness of support items (face mask)	3.92 ± 1.08
Process	Operation of program	Responsiveness to calls	3.99 ± 0.86
		Appropriate consultation time	4.03 ± 0.87
		Easy-to-understand explanations from doctors	4.24 ± 0.75
		Timeliness of support items (educational brochure, mask)	3.64 ± 1.15
Product (output)	Effectiveness	Program effectiveness	3.95 ± 0.91
	Satisfaction	Satisfaction with phone consultation	4.10 ± 0.86
		Satisfaction with educational brochure	3.89 ± 0.87
		Satisfaction with program	3.86 ± 0.94
	Impact of program	Achievement of program goals	3.91 ± 0.90

* Mean ± standard deviation.

**Table 6:** Areas affecting satisfaction with the Follow-up Health Consultation Program.

Area	*β* ± SE	*p*-Value *	R^2^
Context	0.09 ± 0.06	0.456	0.71
Input	0.31 ± 0.11	< 0.001	
Process	0.15 ± 0.09	< 0.001	

* *p*-values calculated using multiple regression analysis. SE, standard errors.

## Data Availability

Data that are not presented in the article are available upon reasonable request from the corresponding author.

## Supplementary Materials

The following supporting information can be downloaded at: https://www.mdpi.com/article/10.3390/ijerph19137996/s1, S1: Questionnaire for doctor; S2: Questionnaire for patient.

## References

[1] Lewnard J.A., Lo N.C. (2020). Scientific and Ethical Basis for Social-Distancing Interventions Against COVID-19. Lancet Infect. Dis..

[2] Koh W.C., Naing L., Wong J. (2020). Estimating the Impact of Physical Distancing Measures in Containing COVID-19: An Empirical Analysis. Int. J. Infect. Dis..

[3] Thunström L., Newbold S.C., Finnoff D., Ashworth M., Shogren J.F. (2020). The Benefits and Costs of Using Social Distancing to Flatten the Curve for COVID-19. J. Benefit Cost Anal..

[4] Torales J., O’Higgins M., Castaldelli-Maia J.M., Ventriglio A. (2020). The Outbreak of COVID-19 Coronavirus and Its Impact on Global Mental Health. Int. J. Soc. Psychiatry.

[5] Stevens A. (2020). Governments Cannot Just “Follow the Science” on COVID-19. Nat. Hum. Behav..

[6] Kim J.Y., Lee Y.M., Lee H., Kim J.W., Kim S.W. (2021). Epidemiological Characteristics of a COVID-19 Outbreak Caused by Religious Activities in Daegu, Korea. Epidemiol. Health.

[7] Kang H.S., Kim B.N. (2021). The Role of Event-Related Rumination and Perceived Social Support on Psychological Distress during the COVID-19 Pandemic: Results from Greater Daegu Region in South Korea. Psychiatry Investig..

[8] Lee Y., Kim B.W., Kim S.W., Son H., Park B., Lee H., You M., Ki M. (2021). Precautionary Behavior Practices and Psychological Characteristics of COVID-19 Patients and Quarantined Persons. Int. J. Environ. Res. Public Health.

[9] Lee K.M., Ko H.J., Lee G.H., Kim A.S., Lee D.W. (2021). A Well-Structured Follow-up Program Is Required After Recovery from Coronavirus Disease 2019 (COVID-19); Release from Quarantine Is Not the End of Treatment. J. Clin. Med..

[10] Kim Y.A., Lee G.H., Lee K.M., Ko H.J., Lee D., Kim A.S. (2020). Communication and Cooperation Between the Medical Academy, Medical Association, and Local Government: Health Counseling Program after Recovery from Coronavirus Disease 2019 (COVID-19) in Daegu. Front. Public Health.

[11] Abadie A., Cattaneo M.D. (2018). Econometric Methods for Program Evaluation. Annu. Rev. Econ..

[12] Stufflebeam D.L. (1972). The Relevance of the CIPP Evaluation Model for Educational Accountability. SRIS Quart..

[13] Stufflebeam D.L., Madaus G.F., Scriven M., Stufflebeam D.L. (1983). The CIPP Model for Program Evaluation. Evaluation Models.

[14] Stufflebeam D.L., Stufflebeam D.L., Madaus G.F., Kellaghan T. (2000). The CIPP Model for Evaluation. Evaluation Models.

[15] Lee M.P. (2012). Vocational Competency Development Education Program Evaluation and Educational Needs Survey Through CIPP Evaluation Model: The Case of K1 Educational Institution. Int. J. Adult Contin. Educ..

[16] Oche M., Adamu H. (2013). Determinants of Patient Waiting Time in the General Outpatient Department of a Tertiary Health Institution in Northwestern Nigeria. Ann. Med. Health Sci. Res..

[17] Chunara R., Zhao Y., Chen J., Lawrence K., Testa P.A., Nov O., Mann D.M. (2021). Telemedicine and Healthcare Disparities: A Cohort Study in a Large Healthcare System in New York City During COVID-19. J. Am. Med. Inform. Assoc..

[18] Dawson D.L., Golijani-Moghaddam N. (2020). COVID-19: Psychological Flexibility, Coping, Mental Health, and Wellbeing in the UK during the Pandemic. J. Contextual Behav. Sci..

[19] Carriedo A., Cecchini J.A., Fernandez-Rio J., Méndez-Giménez A. (2020). COVID-19, Psychological Well-Being and Physical Activity Levels in Older Adults during the Nationwide Lockdown in Spain. Am. J. Geriatr. Psychiatry.

[20] Holingue C., Kalb L.G., Riehm K.E., Bennett D., Kapteyn A., Veldhuis C.B., Johnson R.M., Fallin M.D., Kreuter F., Stuart E.A. (2020). Mental Distress in the United States at the Beginning of the COVID-19 Pandemic. Am. J. Public Health.

[21] Wang C., Tee M., Roy A.E., Fardin M.A., Srichokchatchawan W., Habib H.A., Tran B.X., Hussain S., Hoang M.T., Le X.T. (2021). The Impact of COVID-19 Pandemic on Physical and Mental Health of Asians: A Study of Seven Middle-Income Countries in Asia. PLoS ONE.

[22] Duan L., Zhu G. (2020). Psychological Interventions for People Affected by the COVID-19 Epidemic. Lancet Psychiatary.

[23] Kim B.H. (2018). The Evaluation of Day Care Center In-Service Education Program Using the CIPP Evaluation Model. J. Korea Acad. Ind. Coop. Soc..

[24] Mohebbi N., Akhlaghi F., Yarmohammadian M.H., Khoshgam M. (2011). Application of CIPP Model for Evaluating the Medical Records Education Course at Master of Science Level at Iranian Medical Sciences Universities. Procedia Soc. Behav. Sci..

[25] Zhang G., Zeller N., Griffith R., Metcalf D., Williams J., Shea C., Misulis K. (2011). Using the Context, Input, Process, and Product Evaluation Model (CIPP) as a Comprehensive Framework to Guide the Planning, Implementation, and Assessment of Service-Learning Programs. J. Higher Educ. Outreach Engagem..

