# Supplementary material for: Evaluation of a Follow-Up Health Consultation Program for Patients with Coronavirus Disease 2019 in Korea: Using the Context–Input–Process–Product Model
Source: Int J Environ Res Public Health. 2022 Jun 29;19(13):7996. doi: 10.3390/ijerph19137996 (PMC9266060; doi:10.3390/ijerph19137996)
Supplement: Supplementary file 1 [file ijerph-19-07996-s001.zip › S1 Appendix_Questionnaire for doctor.pdf]

S1 Appendix

The Follow-Up Health Consultation Program for COVID-19 patients Evaluation Survey

: for Doctor

We sincerely thank you for your active participation and volunteering in the follow-up health consultation program for those who have recovered from COVID-19 jointly conducted by the Daegu-Gyeongbuk Branch of the Korean Association of Family Medicine and Daegu City. We would like to give an overall evaluation of the program. The results of this survey will serve as an opportunity to improve the program for patients and explore the role of family medicine specialists in the future of infectious disease epidemics. The results of this survey are used only for administrative and academic purposes, and no personal information is collected. It is expected that it will take about 20 minutes to answer the questionnaire. Thank you for all your hard work.

Please select whether or not to participate in this survey.

□ Agree □ Disagree

1. Demographics

The following questions ask about your basic personal information. Please answer the applicable questions.

1-1. Age (   )

1-2. Sex □ Male   □ Female

1-3. Year of acquisition of specialist (   )

1-4. Work place/type

□ Individual practice   □ Group practice   □ Local hospital   □ Teaching hospital

2. Administrative matters.

The following questions are about the administrative matters of the consultation process. Please answer the applicable questions.

2-1. Was the consultation conducted quickly?

□ Definitely   □ Probably   □ Possibly   □ Probably Not □ Definitely Not

2-2. How long was the average duration of one consultation?

□ within 10 minutes   □ 11-20 minutes   □ 21-30 minutes   □ 31 minutes or more

3. Evaluation for context area

The following is an assessment of the context in the program. Please answer the applicable questions.

3-1. Do you think the goals of this program are clearly stated?

□ Definitely   □ Probably   □ Possibly   □ Probably Not □ Definitely Not

3-2. Do you think the goals of this program were appropriate?

□ Definitely   □ Probably   □ Possibly   □ Probably Not □ Definitely Not

3-3. Did you think the goals of this program would be achievable?

□ Definitely   □ Probably   □ Possibly   □ Probably Not □ Definitely Not

4. Evaluation for input area

4-1. Was the number of assigned patients adequate to conduct the consultation?

□ Definitely □ Probably □ Possibly □ Probably Not □ Definitely Not

4-2. Do you think the counseling support items (educational brochure, face mask) were helpful in the operation of this program?

□ Definitely □ Probably □ Possibly □ Probably Not □ Definitely Not

5. Evaluation for process area

5-1. Was the time appropriate for the call to be connected to the patients? (Did the patients answer the phone well?)

□ Definitely □ Probably □ Possibly □ Probably Not □ Definitely Not

5-2. How was the response to the phone consultations of patients?

□ Definitely □ Probably □ Possibly □ Probably Not □ Definitely Not

5-3. Did the health consultation for a patients interfere with your main work?

□ Definitely □ Probably □ Possibly □ Probably Not □ Definitely Not

5-4. Did the health consultation for a patients interfere with your personal time outside of work?

□ Definitely □ Probably □ Possibly □ Probably Not □ Definitely Not

6. Evaluation for output area

6-1. Do you think this program was useful?

□ Definitely □ Probably □ Possibly □ Probably Not □ Definitely Not

6-2. Are you satisfied with this program as a doctor?

□ Definitely □ Probably □ Possibly □ Probably Not □ Definitely Not

6-3. Do you think this program has achieved its intended goals?

□ Definitely □ Probably □ Possibly □ Probably Not □ Definitely Not

7. Overall evaluation of the program

Please feel free to answer the following questions about your overall evaluation of the program.

7-1. What was the good thing about this program? (Please write freely)

7-2. Were there any problems during the implementation of the program? What problems did you have? How did you deal with the problem? (Please write freely)

7-3. Is there anything that needs improvement about this program? (Please write freely)

7-4. If you have any thoughts or opinions while conducting the program, please feel free to describe them.
