# Supplementary material for: Evaluation of a Follow-Up Health Consultation Program for Patients with Coronavirus Disease 2019 in Korea: Using the Context–Input–Process–Product Model
Source: Int J Environ Res Public Health. 2022 Jun 29;19(13):7996. doi: 10.3390/ijerph19137996 (PMC9266060; doi:10.3390/ijerph19137996)
Supplement: Supplementary file 1 [file ijerph-19-07996-s001.zip › S2 Appendix_Questionnaire for patient.pdf]

S2 Appendix

The Follow-Up Health Consultation Program for COVID-19 patients Evaluation Survey

: for Patient

We sincerely thank you for your participation in the follow-up health consultation program for those who have recovered from COVID-19 jointly conducted by the Daegu-Gyeongbuk Branch of the Korean Association of Family Medicine and Daegu City. We hope this program has been helpful to you in managing your health. We would like to give an overall evaluation of the program. The results of this survey will serve as an opportunity to improve the program for patients. The results of this survey are used only for administrative and academic purposes, and no personal information is collected. It is expected that it will take about 20 minutes to answer the questionnaire. Thank you for all your hard work.

Please select whether or not to participate in this survey.

□ Agree □ Disagree

1. Demographics

The following questions ask about your basic personal information. Please answer the applicable questions.

1-1. Age (   )

1-2. Sex □ Male   □ Female

1-3. Family composition

□ Single □ Married □ 2 or over generation □ Other

1-4. Comorbidity

□ None □ 1 □ 2 or more

1-5. What do you usually do when you have a problem? (Multiple choices are possible)

□ Solve it alone □ Consult with family □ Consult with friends

□ Consult with professional consultant □ Other

2. Other general matters.

The following questions are about the other general matters of the consultation process. Please answer the applicable questions.

2-1. What facility have you been quarantined and treated?

□ Residential treatment center □ Medical institution □ Intensive care unit □ Other

2-2. How long have you been hospitalized or quarantined?

□ 1-7 days □ 8-14 days □ 15-21 days □ 22-28 days □ 29 days or over

2-3. How stressful has this Sars-CoV-2 infection been for you? if you give me a score?

□ 5 (Very stressful) □ 4 □ 3 □ 2 □ 1 □ 0 (No stress at all)

2-4. Was the consultation conducted quickly?

□ Definitely □ Probably □ Possibly □ Probably Not □ Definitely Not

3. Evaluation for context area

The following is an assessment of the context in the program. Please answer the applicable questions.

3-1. Were you well informed about the purpose of the program at the beginning?

3-2. □ Definitely □ Probably □ Possibly □ Probably Not □ Definitely Not

4. Evaluation for input area

4-1. Was your consultant (doctor) professional??

□ Definitely □ Probably □ Possibly □ Probably Not □ Definitely Not

4-2. Were the educational brochures useful to you?

□ Definitely □ Probably □ Possibly □ Probably Not □ Definitely Not

4-3. Were the face masks useful to you?

□ Definitely □ Probably □ Possibly □ Probably Not □ Definitely Not

5. Evaluation for process area

5-1. Did the consultant have a good connection to counseling when I needed it?

□ Definitely □ Probably □ Possibly □ Probably Not □ Definitely Not

5-2. Did your consultant explain to you in a way you could understand?

□ Definitely □ Probably □ Possibly □ Probably Not □ Definitely Not

5-3. Did the health consultation for a patients interfere with your main work?

□ Definitely □ Probably □ Possibly □ Probably Not □ Definitely Not

5-4. Was the support items(educational brochure, face mask) delivered to you on time?

□ Definitely □ Probably □ Possibly □ Probably Not □ Definitely Not

6. Evaluation for output area

6-1. Do you think this program was useful?

□ Definitely □ Probably □ Possibly □ Probably Not □ Definitely Not

6-2. Are you satisfied with the phone consultation?

□ Definitely □ Probably □ Possibly □ Probably Not □ Definitely Not

6-3. Are you satisfied with the educational brochure?

□ Definitely □ Probably □ Possibly □ Probably Not □ Definitely Not

6-4. Are you satisfied with this program?

□ Definitely □ Probably □ Possibly □ Probably Not □ Definitely Not

6-5. Do you think this program has achieved its intended goals?

□ Definitely □ Probably □ Possibly □ Probably Not □ Definitely Not

7. Overall evaluation of the program

Please feel free to answer the following questions about your overall evaluation of the program.

7-1. What was the good thing about this program? (Please write freely)

7-2. Is there anything that needs improvement about this program? (Please write freely)

7-3. If you have any thoughts or opinions while conducting the program, please feel free to describe them.
